# Supplementary material for: Stacking Multiple Genes Improves Resistance to Chilo suppressalis, Magnaporthe oryzae, and Nilaparvata lugens in Transgenic Rice
Source: Genes (Basel). 2023 May 12;14(5):1070. doi: 10.3390/genes14051070 (PMC10218540; doi:10.3390/genes14051070)
Supplement: Supplementary file 1 [file genes-14-01070-s001.zip › genes-2269945-supplementary.pdf]

Supplementary Materials

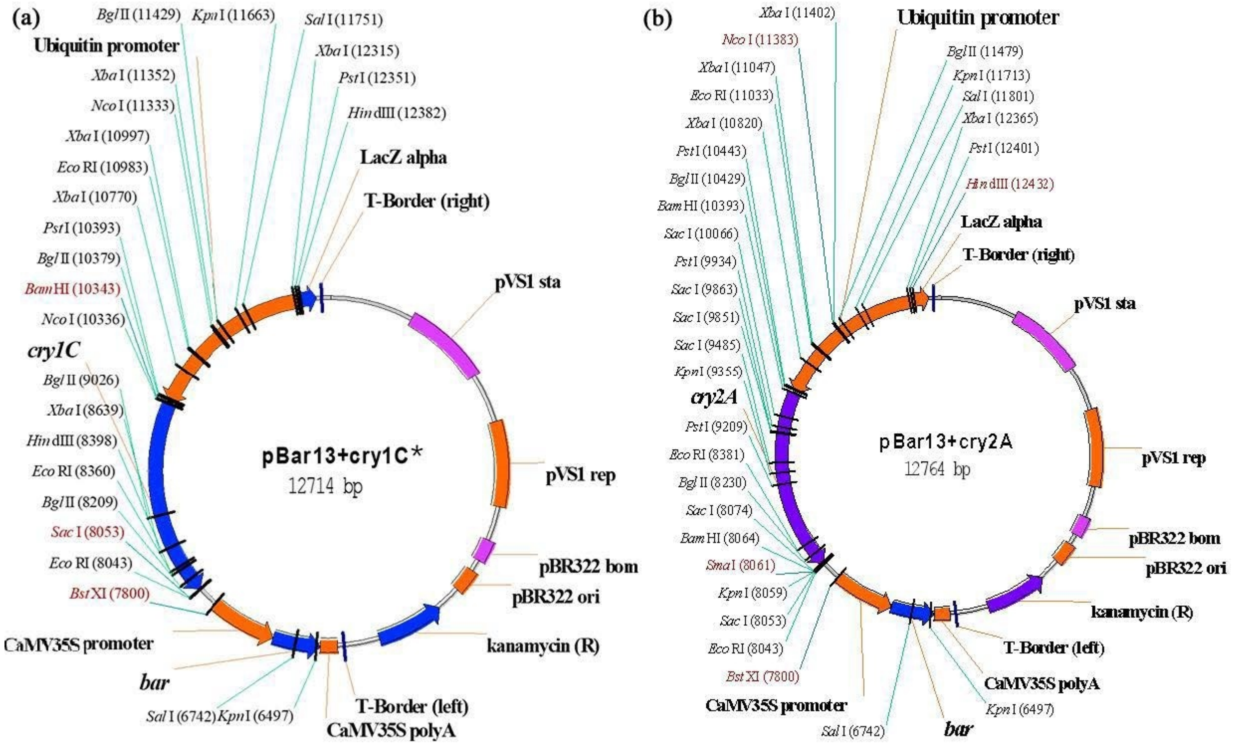

Figure S1. Construction of *CRY1C* and *CRY2A* vectors. The *CRY1C* and *CRY2A* gene was driven by a Ubiquitin promoter and terminated by the nopaline synthase (Nos) terminator. T-Border (left): left border of T-DNA region; T-Border (right): right border of T-DNA region.

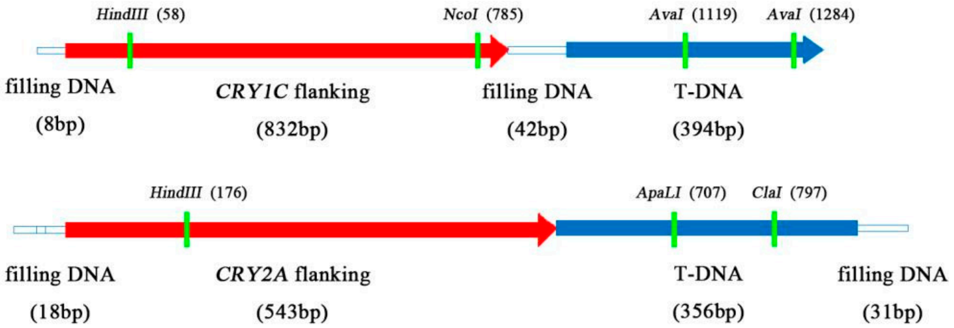

Figure S2. Schematic map of flanking sequences of *CRY1C* and *CRY2A* insertion site.

Table S1 PCR primers used in this study

Gene	Primer	Primer sequence(5'-3')	Gene length	Tm(℃)
CRY 1C	CRY 1C-F	TTCTACTGGGGAGGACATCG	799bp	57
	CRY 1C-R	CGGTATCTTGGGTGATTGG		

CRY 2A	CRY 2A-F	CGTGTCAATGCTGACCTGAT	600bp	58
	CRY 2A-R	GATGCCGGACAGGATGTAGT		
Pib	Pibgom-F	GAACAATGCCCAAACTTGAGA	360bp	55
	Pibgom-R	GGGTCCACATGTCAGTGAGC		
	Lys145-F	TCGGTGCCTCGGTAGTCAGT	800bp	61
	Lys145-R	GGGAAGCGGATCCTAGGTCT		
Pikm	DKm1-F	CTGGAGAGCTTCCGTGTCGAC	191bp	60
	DKm1-R	TCTTCACGACGTCAATGGTGGC		
	DKm2-F	GTTGTTCACTCCGTATCTACTACGTC	223bp	60
	DKm2-R	TTCCTCCGTGATCACAGCAACG		
Bph29	Bph29-F	GACACCGTCTCCTTCTGCC	570bp	56
	Bph29-R	TTGCTTCCATCGTCCAATT		

Table S2 Physical locations of molecular markers

**Chr**	**Chromosome length/Mb**	**Marker_num**	**Average length between markers/Mb**
chr1	435.10	58	7.50
chr2	358.50	58	6.18
chr3	363.00	57	6.37
chr4	351.80	49	7.18
chr5	296.70	43	6.90
chr6	309.80	43	7.20
chr7	295.60	47	6.29
chr8	280.70	46	6.10
chr9	229.20	35	6.55
chr10	227.50	40	5.69
chr11	284.00	41	6.93
chr12	273.00	43	6.35

Table S3 Resistant level criteria of *Chilo suppressalis*

**Corrected larval mortality（%）**	**Resistance level**
85-100	Highly Resistant(HR)
60-85	Resistant(R)
20-60	Moderately Resistant(MR)
0-20	Sensitive(S)

Table S4 PDA medium formula and rice bran medium

Type	Formula
PDA medium	Potato 200g, sucrose 20g, agar 20g,Dd H_2O 1000ml
Rice bran medium	Rice bran 20g ,agar 20g , yeast extract 2.5g,Dd H_2O 1000ml

Table S5 Identification of resistance to *Magnaporthe oryzae* criteria

Situation at onset	Resistance level
Without infection or any scab	Highly Resistant(HR)
With scattered brown spots less than 0.5 mm in diameter.	Resistant(R)
Exist a few brown spots with a diameter of about 0.5 to 1 mm.	Moderately Resistant(MR)
Round to elliptical lesions less than 3 mm in diameter were present; Central gray-white, margin brown.	Moderately Sensitive(MS)
Typical spindle-shaped rice blast spot, diameter 3mm or more, with slightly fused or unfused plaques.	Sensitive(S)
Typical spindle-shaped rice blast spot, diameter 3mm or longer, spots confluently confluent into sheets, resulting in the apical or integral death of the leaf blade.	Highly Sensitive(HS)

Table S6 Identification of resistance to *Nilaparvata lugens* criteria

Seedling mortality(%)	Resistance level
＜1.0	Immune(I)
1.1-10.0	Highly Resistant(HR)
10.1-30.0	Resistant(R)
30.1-50.0	Moderately Resistant(MR)
50.1-70.0	Sensitive(S)
>70.1	Highly Sensitive(HS)

Table S7 Measurement of agronomic traits investigation method

Agronomic traits	Methods of investigating

Plant height (cm)	The distance from the ground to the highest ear tip per plant
Effective panicle number	Number of ears with more than 10 seeds per plant
Panicle length(cm)	Length from ear neck to ear tip
Total grain number	Total grain number/effective ear number per plant
Number of solid grains	Grain number per plant/effective ear number per plant
Seed setting rate(%)	Grain number per plant/total grain number per plant
1000-grain weight(g)	(seed weight per plant/seed number per plant) ×1000
Yield per plant(g)	Weight of all grains per plant

Table S8 Response rate of each strains

Transgenic restorer line	Number of SSR markers	Number of polymorphic SSR marker	Polymorphic marker rate (%)	Theoretical response rate (%)	Actual response rate (%)
CH121TJH	560	53	9.46	83.34	85.85
CH891TJH	560	49	8.75	83.34	90.82
R205XTJH	560	55	9.82	83.34	89.09

Table S9 *C. suppressalis* corrected larval mortality rate

Variety	R.gene	Number of dead larval	Mortality rate(%)	Corrected mortality rate(%)	Resistance level
CH121	/	2.00	6.67	/	Sensitive(S)
CH121TJH	*CRY 1C,Pibgom,Pikm,Bph29*	26.67	88.90	88.11	Highly Resistant(HR)
CH891	/	1.67	5.57	/	Sensitive(S)
CH891TJH	*CRY 2A,Pibgom,Pikm,Bph29*	25.00	83.33	82.35	Resistant(R)
R205X	/	1.33	4.33	/	Sensitive(S)
R205XTJH	*CRY 2A,Pibgom,Pikm,Bph29*	24.67	82.23	81.41	Resistant(R)

Table S10 The *M. oryzae* diseased spot accounts for the leaf area

Variety	Area 1	Area 2	Area 3
LTH	83%	80%	89%
CK30	0	0	0
CH121	30%	35%	28%
CH891	25%	25%	27%
R205X	20%	25%	30%
CH121TJH	8%	6%	5%
CH891TJH	0	1%	1%

R205XTJH	5%	7%	3%

Table S11 Mortality rate in assessment of *N. lugens* resistance at the seedling stage

Variety	Number of dead seedlings	Number of total seedlings	mortality rate(%)	Resistance level
CH121	24.67±2.49	28	88.09±8.91	Highly Sensitive(HS)
CH121TJH	2.33±2.05	28	8.33±2.34	Highly Resistant(HR)
CH891	18.33±1.25	28	65.48±4.46	Sensitive(S)
CH891TJH	5.33±1.25	28	19.05±4.45	Resistant(R)
R205X	14.33±1.25	28	51.19±4.45	Sensitive(S)
R205XTJH	7.33±1.25	28	26.19±4.45	Resistant(R)
PSL	0.00	28	0.00	Immune(I)
TN1	26.67±1.25	28	94.05±3.37	Highly Sensitive(HS)
